# The Roles of EphB2 in Cancer

**DOI:** 10.3389/fcell.2022.788587

**Published:** 2022-02-10

**Authors:** Wei Liu, Chengpeng Yu, Jianfeng Li, Jiwei Fang

**Affiliations:** ^1^ Department of Geriatrics, The First Affiliated Hospital of Nanchang University, Nanchang, China; ^2^ Second Clinical Medical College, Nanchang University, Nanchang, China; ^3^ Department of General Surgery, The First Affiliated Hospital of Nanchang University, Nanchang, China

**Keywords:** receptor tyrosine kinase, EphB2 receptor, cancer, tumor progression, biomarker

## Abstract

The erythropoietin-producing hepatocellular carcinoma (Eph) receptors and their Eph receptor-interacting (ephrin) ligands together constitute a vital cell communication system with diverse roles. Experimental evidence revealed Eph receptor bidirectional signaling with both tumor-promoting and tumor-suppressing activities in different cancer types and surrounding environment. Eph receptor B2 (EphB2), an important member of the Eph receptor family, has been proved to be aberrantly expressed in many cancer types, such as colorectal cancer, gastric cancer and hepatocellular carcinoma, resulting in tumor occurrence and progression. However, there are no reviews focusing on the dual roles of EphB2 in cancer. Thus, in this paper we systematically summarize and discuss the roles of EphB2 in cancer. Firstly, we review the main biological features and the related signaling regulatory mechanisms of EphB2, and then we summarize the roles of EphB2 in cancer through current studies. Finally, we put forward our viewpoint on the future prospects of cancer research focusing on EphB2, especially with regard to the effects of EphB2 on tumor immunity.

## Introduction

The erythropoietin-producing hepatocellular carcinoma (Eph) receptors constitute the largest sub-family of receptor tyrosine kinases (RTKs) identified until now (Eph Nomenclature Committee, 1997). The Eph receptors have diverse activities, including effects on the actin cytoskeleton, cell attachment, cell shape, and cell mobility. Moreover, recent work has also found that these receptors also influence cell proliferation, survival, secretion, and differentiation. These activities depend on the interaction between the Eph receptors and the ephrins (Eph receptor interacting proteins) (Pasquale, 2005; Pasquale, 2008). Based on sequence identity, structure, and their binding affinity for ligands, the Eph receptors are grouped into two subclasses, EphA receptor (EphA1-10) and EphB receptor (EphB1–6). The ligands for Eph receptors, ephrins, are cell-surface bound proteins that are divided into two subclasses ephrin-A (ephrin-A1 to -A6) and ephrin-B (ephrin-B1 to -B3) according to how they bind to the plasma membrane (Pasquale, 2004; Pasquale, 2005). Ephrin-A ligands are glycosylphosphatidylinositol (GPI) anchored, and ephrin-A ligands can transmit signals despite the lack of a cytoplasmic domain. The reverse signaling mechanisms of ephrin-A ligands are considered to be related to ephrin-A clustering and recruitment of regulatory proteins (Davy et al., 1999). Ephrin-A ligands are anchored to the membrane via covalent linkage to GPI, and rely on transmembrane coreceptors to transmit signals intracellularly (Bonanomi et al., 2012). Ephrin-B ligands are similar to Eph receptors in that they contain a cytoplasmic region, a single transmembrane domain, and a PDZ-binding motif. Ephrin-B reverse signaling also involves the Src family kinases, which are responsible for ephrin-B phosphorylation after the binding of Eph receptor (Pasquale, 2005; Arvanitis and Davy, 2008). Phosphorylated ephrin-B can initiate reverse signaling via SH2 or PDZ domain-containing proteins (Cowan and Henkemeyer, 2001; Lu et al., 2001). In general, EphA receptors are promiscuously activated by glycosylphosphatidylinositol (GPI)-linked ephrin-A ligands, and EphB receptors are promiscuously activated by transmembrane ephrin-B ligands. However, there are some exceptions, cross interactions have been observed between EphA4 and ephrin-B2/B3 as well as between EphB2 and ephrin-A5 (Himanen et al., 2004; Kania and Klein, 2016; Royet et al., 2017). Furthermore, the membrane attachment of both Eph receptors and ephrin ligands provides a mechanism whereby Eph-ephrin receptors signaling activation requires cell-cell contacts. Eph receptors interact with their membrane-bound ligands the ephrins and promote cell-cell contacts, leading to bidirectional intracellular signaling and downstream signaling cascades that induce autophosphorylation of tyrosine residues in the juxtamembrane region and kinase domain, which further drive the recruitment of downstream signaling molecules (Kullander and Klein, 2002). These include Src family kinases, mitogen-activated protein (MAP) kinases, Src homology 2 and 3 adapter proteins, guanine nucleotide exchange factors, phosphatidylinositol 3-kinase (PI3K), small GTPases, and phosphatases. They are all involved in complex cell-cell repulsion and adhesion pathways, which modulate cell morphology, motility and attachment (Pasquale, 2005).

It is widely known that Eph receptors have essential roles in embryonic development, and in the past decade their critical roles in the occurrence and progression of human disease, especially in tumorigenesis, have become more and more clear (Pasquale, 2008; Xi et al., 2012; Husa et al., 2016). Undoubtedly, enriching our understanding of the roles Eph receptors play in the potential biomarkers, stemness, and drug resistance of cancer will provide new opportunities for tumor therapy (Pasquale, 2010; Leung et al., 2021). Eph receptor B2 (EphB2) has been demonstrated to play a crucial modulatory role in tumor progression (Guo et al., 2006; Lam et al., 2014; Buckens et al., 2020; Morales et al., 2021). Perplexingly, EphB2 can function as both tumor promoters and suppressors in different cellular contexts. In many different human tumors, such as breast cancer, cervical cancer, and medulloblastoma (Wu et al., 2004; Sikkema et al., 2012; Duan et al., 2018), EphB2 acts as a tumor promoter that promotes migration and invasion of tumor cells, and its expression is upregulated. On the contrary, in colorectal cancer and bladder cancer (Xi et al., 2012; Lee et al., 2021), EphB2 functions as a tumor suppressor and its expression level is reduced. However, there are no reviews focusing on the dual roles of EphB2 in cancer. Hence, in the subsequent chapters we first review the main biological features and the related signaling regulatory mechanisms of EphB2, and then we summarize the roles of EphB2 in cancer through current studies, in order to provide some fundamental knowledge for following studies. Finally, we provide our viewpoint on the future prospects of cancer research focusing on EphB2, especially with regard to the effects of EphB2 on tumoral immunity.

## The Biological Features of EPHB2

**FIGURE 1:**
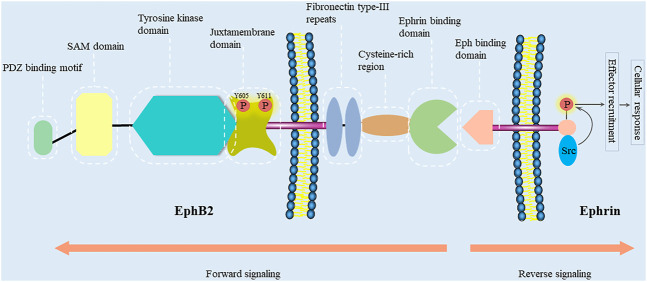
EphB2 and ephrin domain structure and their interacting proteins. Eph receptors contain an N-terminal multidomain extracellular region, a membrane spanning region, and an intracellular region. The intracellular region encompasses a juxtamembrane region, a tyrosine kinase domain, a sterile-α-motif (SAM) domain, and a PDZ-binding motif. The extracellular region includes two fibronectin type-III repeats, a cysteine-rich domain (containing an epidermal growth factor (EGF)-like motif), and an ephrin-binding region. Bidirectional signaling causes forward signaling via Eph receptors and reverse signaling via ephrin ligands. The cellular response caused by Eph/ephrin reverse signaling depends on the intracellular environment. In general, ephrin-B binding of EphB receptors results in the recruitment of Src family kinase and the phosphorylation of the intracellular region of ephrin-B (Kania and Klein, 2016).

The EphB2 receptor is a 117-kDa transmembrane protein consisting of 1,055 amino acids, which is encoded on chromosome 1p36.12 in humans. It was cloned from chicken cDNA in 1991 (Pasquale, 1991). EphB2 has a prototypical RTK topology including an N-terminal multidomain extracellular region, a membrane spanning region, and an intracellular region (Figure 1) (Beckmann et al., 1994; Lisabeth et al., 2013). The extracellular region includes two fibronectin type-III repeats, a cysteine-rich domain (containing an epidermal growth factor (EGF)-like motif), and an ephrin-binding region (Himanen et al., 2001; Pasquale, 2005). The ephrin-binding region of EphB2 is a spherical ligand-binding region containing a cavity that accommodates a hydrophobic loop protruding from the ephrin (Himanen et al., 2001). Interestingly, so as to accommodate to ephrin-A5 or ephrin-B2, different conformational changes occur in the ligand-binding cavity of EphB2 (Toth et al., 2001; Himanen et al., 2004). Moreover, EphB2 can also bind ephrin-B1 and ephrin-B3 (Tanaka et al., 2007; McClelland et al., 2010).

The intracellular region encompasses a juxtamembrane region, a tyrosine kinase domain, a sterile-α-motif (SAM) domain, and a PDZ-binding motif (Pasquale, 2008). The SAM domain is a protein interaction domain that facilitates receptors homo-dimerization and the oligomerization (Thanos et al., 1999). Moreover, biophysical and structural studies have revealed that isolated extracellular Eph and ephrin regions initially form high-affinity heterodimers around the hydrophobic loop of the ligand. Then, these dimers may form ring-like heterotetramers with lower affinity. On cell surface, Eph-ephrin complexes are further arranged into higher-order aggregates or clusters, and begin a bidirectional signaling (Himanen et al., 2001; Himanen and Nikolov, 2002; Himanen, 2012). EphB2 signals may also be propagated through the Ras binding protein AF6 and other proteins containing the SH2 domain, which bind to the C-terminus of the Eph receptors (Hock et al., 1998; Torres et al., 1998; Buchert et al., 1999). Furthermore, there are two conserved autophosphorylation sites (tyrosines 605 and 611) in the EphB2 juxtamembrane region (Zisch et al., 2000). In order to investigate their role in Src binding, Zisch et al. have mutated tyrosines 605 and 611 of EphB2 to the amino acid phenylalanine that cannot be phosphorylated (Zisch et al., 1998). However, replacing tyrosines 605 and 611 with phenylalanine reduce EphB2 kinase activity, which complicates the analysis of their role in EphB2-mediated signaling and their function as Src homology 2 (SH2) domain binding sites. In contrast, replacing them with glutamic acid, which like phosphotyrosine is negatively charged, will disrupt SH2 domain binding without impairing EphB2 kinase activity (Zisch et al., 2000). Functionally, Ephb2 is related to monocyte adhesion to endothelial cells (Vreeken et al., 2020), endothelial cell chemotaxis and branching remodeling (Salvucci et al., 2006), T-cell and B-cell activation (Nguyen et al., 2013; Yu et al., 2014; Mimche et al., 2015a), autophagic cell death (Kandouz et al., 2010; Tanabe et al., 2011), cell repulsive responses (Lin et al., 2008; Poliakov et al., 2008; Schaupp et al., 2014; Gaitanos et al., 2016; Okumura et al., 2017; Evergren et al., 2018), platelet function (Vaiyapuri et al., 2015; Berndt and Andrews, 2018; Berrou et al., 2018), angiogenesis (Sato et al., 2019), and liver fibrosis (Mimche et al., 2015b; Butler and Schmidt, 2015; Mimche et al., 2018; Chen et al., 2020; Huang et al., 2021).

## Forward and Reverse Signaling

A distinctive characteristic of Eph-ephrin complexes is that they can generate bidirectional signals: Eph/ephrin forward signaling is triggered by activating tyrosine kinase domain after the binding of ephrin ligand, and propagates in the receptor-expressing cells, whereas Eph/ephrin reverse signaling is initiated by activating Src family kinase domain after the binding of Eph receptor, and propagates in the ligand (ephrin)-expressing cells (Surawska et al., 2004; Pasquale, 2010). Eph signaling modifies the actin cytoskeleton organization and affects the activities of intercellular adhesion molecules and integrins, thereby controlling cell morphology, adhesion, invasion, migration, and the epithelial phenotype (Pasquale, 2005; Pasquale, 2008). In addition, recent work has also discovered Eph influences on cell proliferation, survival, and special cellular functions such as insulin secretion, immune function, synaptic plasticity and bone remodeling (Pasquale, 2005).

Regarding forward signaling by EphB2 in cancer cells, although the receptor is upregulated in most cancers, its response to ephrin is silent. In some cases, Eph forward signaling that relies on ephrin may even be harmful to tumor progression. For example, the medulloblastoma cell lines with high expression of EphB2 were stimulated by ephrin-B1, the cell adhesion ability *in vitro* was significantly decreased, and the invasion ability was increased (Sikkema et al., 2012). At the same time, overexpression of EphB2 in glioma tissues and cells inhibited cell adhesion and promoted cell invasion, indicating that the overexpression of EphB2 promotes tumor progression via forward signaling (Nakada et al., 2004; Nakada et al., 2005). However, EphB2 inactivation promoted cell proliferation, motility, and invasion of bladder cancer (Lee et al., 2021). Silencing EphB2 accelerated pancreatic cancer growth by facilitating cell proliferation through triggering G1/S phase transition, indicating EphB2 forward signaling has a tumor suppressing function (Hua et al., 2011).

Unlike Eph receptors, since ephrin-Bs do not have intrinsic catalytic activity, they depend on the recruitment of signaling molecules (such as Src family kinases) to signal, which phosphorylate specific tyrosine residues in the cytoplasmic region of ephrin-Bs, leading to receptor engagement and clustering (Salvucci and Tosato, 2012; Salgia et al., 2018). Moreover, similar to the case of forward signaling, reverse signaling was also found to lead to tumor progression and suppression. For example, EphB2-ephrin-B1 promoted the invasion of pancreatic cancer cells (Tanaka et al., 2007). However, EphB2/ephrin signaling was able to suppress colorectal cancer expansion and invasion via repulsive mechanisms (Okumura et al., 2017; Evergren et al., 2018). In summary, these results on bidirectional signaling indicated that EphB2/ephrin has diverse and complex functions in different cancer types and surrounding environment. In addition, mutational inactivation of EPHB2 may also play an important role in cancer progression (Huusko et al., 2004).

## EPHB2 in Various Human Tumors

**FIGURE 2:**
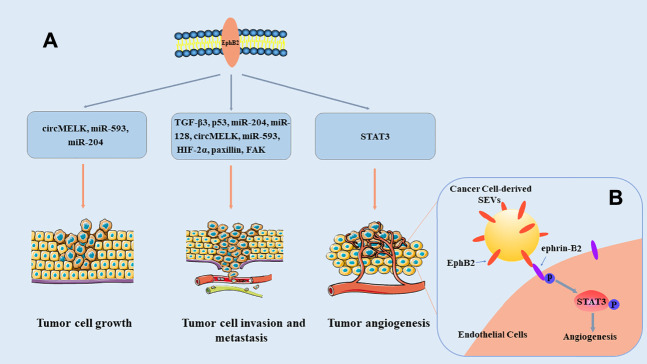
Overview of the roles of EphB2 in cancer. **(A)** EphB2 exerts its roles in tumor cell growth, invasion and metastasis, as well as angiogenesis through multiple signaling pathways. **(B)** Model of SEV-induced angiogenesis pathway. EphB2 on cancer cell–derived small extracellular vesicles (SEVs) binds to ephrin-B2 on endothelial cells, and induces ephrin-B2 reverse signaling through downstream phosphorylation and activation of STAT3, thereby promoting angiogenesis (Sato et al., 2019).

Many studies have verified that EphB2 is abnormally expressed in many cancer types Table 1. EphB2 is overexpressed in most tumors, such as hepatocellular carcinoma, breast cancer, glioma, and malignant mesothelioma (Leung et al., 2021; Wu et al., 2004; Nakada et al., 2004; Goparaju et al., 2013), and it functions as a tumor promoter. However, the expression of EphB2 is low in other tumors, such as colorectal cancer and bladder cancer (Xi et al., 2012; Lee et al., 2021), indicating that it exerts a tumor-suppression effect. These studies show that EphB2 expression is dynamically regulated in different tumor progression, and that EphB2 exerts its regulatory functions in multiple ways (Figure 2). Moreover, EphB2 expression is related to elevated metastatic potential, poor prognosis, and decreased survival of tumor patients (Wu et al., 2006; Yu et al., 2011; Husa et al., 2016; Koh et al., 2020). Consequently, EphB2’s functional relevance and expression patterns in malignancies make this protein a potential prognostic biomarker and therapeutic target in cancer.

**TABLE 1:** The expression levels and functions of EphB2 in different tumors.

Cancer type	EphB2 expression	Related proteins	Involved signaling pathways	Associated cellular process	Clinicopathological features	References
Gastric cancer	Upregulated	—	JAK-STAT and TP53 signaling	Promotes migration, invasion, and inhibits adhesion	Poorer overall survival	Kataoka et al. (2002); Yin et al. (2020)
	Downregulated	—	—	—	Lymph node metastasis, advanced T stage, poorer histological differentiation, poorer overall survival	Yu et al. (2011)
Prostate cancer	Downregulate; mutational inactivation	DGAT1; ATGL	—	Inhibits cell proliferation, invasion, and intracellular lipid accumulation	—	Huusko et al. (2004); Morales et al. (2021)
	Upregulated	—	—	Promotes cell proliferation, migration, invasion	—	Liu et al. (2019)
Colorectal cancer	Downregulated	c-Rel	TCF/β-catenin signaling	Inhibits migration, invasion	Higher histological tumor grade, poorer differentiation, poorer overall survival and disease-free survival	Batlle et al. (2002); Guo et al. (2006); Fu et al. (2009); Senior et al. (2010)
Breast cancer	Upregulated	TGF-β3; p53	—	Promotes migration, invasion	Poorer overall survival and disease-free survival	Wu et al. (2004); Lam et al. (2014); Husa et al. (2016)
Hepatocellular carcinoma	Upregulated	TCF1	Wnt/β-catenin signaling	-	Poorer overall survival and disease-free survival	Leung et al. (2021)
Pancreatic cancer	Upregulated	—	—	—	Lymph node metastasis, higher degree of pain, poorer overall survival	Lu et al. (2012); Chen et al., (2019)
Cutaneous squamous cell carcinoma	Upregulated	MMP1; MMP13	—	Promotes cell proliferation, migration, invasion, and angiogenesis	—	Farshchian et al. (2015)
Head and neck squamous cell carcinoma	Upregulated	STAT3	—	Promotes angiogenesis	Poorer overall survival	Sato et al. (2019)
Glioma	Upregulated	miR-204; miR-128	—	Promotes migration, invasion, and inhibits adhesion	Higher tumor grade	Nakada et al. (2004); Lin et al. (2013); Ying et al. (2013)
Glioblastoma multiforme	Upregulated	HIF-2α; circMELK; miR-593; paxillin	—	Promotes cell proliferation, migration, invasion	Poorer overall survival	Qiu et al., (2019); Zhou et al., (2021)
Medulloblastoma	Upregulated	Erk; p38; mTOR	—	Promotes migration, invasion, and inhibits adhesion	—	Sikkema et al. (2012); Bhatia et al. (2017)
Cervical cancer	Upregulated	miR-204	R-Ras signaling	Promotes cell proliferation, migration, invasion	Metastasis	Gao et al. (2014); Duan et al. (2018)
Malignant mesothelioma	Upregulated	VEGF; MMP-2; caspase-2; caspase-8	—	Promotes cell proliferation, migration, invasion, and inhibits apoptosis	—	Goparaju et al. (2013)
Bladder cancer	Downregulated	—	—	Inhibits cell proliferation, invasion	Advanced tumor stage, higher tumor grade, metastasis	Li et al. (2014); Lee et al. (2021)
Wilms tumor	Downregulated	—	—	—	—	Chetcuti et al. (2011)
Cholangiocarcinoma	Upregulated	FAK; paxillin	-	Promotes migration	Metastasis	Khansaard et al. (2014)
Lung adenocarcinoma	Upregulated	—	—	—	Poorer overall survival and disease-free survival	Zhao et al. (2017)
Ovarian carcinoma	Upregulated	—	—	—	Poorer overall survival	Wu et al. (2006)

### EphB2 in Gastric Cancer

EphB2 was found to be overexpressed in gastric cancer (GC) tissues than in adjacent or benign non-cancerous gastric tissues, including gene and protein expression (Kataoka et al., 2002; Yin et al., 2020). EphB2 activation accelerated the invasion and migration abilities of the GC cells. Conversely, EphB2 activation obviously reduced the adhesion in GC cells. Moreover, the enrichment analysis of related genes in a GC cohort showed that EphB2 may play a role by mediating the cytokine-cytokine receptor interaction, TP53 and JAK-STAT signaling pathways (Yin et al., 2020). However, the clinical significance of EphB2 in GC is controversial and contradictory so far (Kataoka et al., 2002; Lugli et al., 2005; Yu et al., 2011). Yu et al. reported that the loss of EphB2 expression in GC was significantly correlated with nodal metastasis and advanced disease stage. As the tumor grade increased, the expression rates of EphB2 lowered significantly. At the same time, univariate and multivariate analysis indicated that the loss of EphB2 expression was significantly associated with poor survival of GC patients (Yu et al., 2011). This further implies that EphB2 also serves as a tumor suppressor in GC. However, the underlying molecular mechanism that can explain this contradictory result needs further research. Furthermore, Davalos et al. found that high mutation rate of EphB2 may be related to microsatellite instability in GC compared with endometrial tumors adopting a limited sample size (Davalos et al., 2007).

### EphB2 in Prostate Cancer

EphB2 expression was frequently found to be decreased in prostate cancer (PC) tissues with somatic mutational inactivation occurred in approximately 10% of sporadic tumors (Huusko et al., 2004; Robbins et al., 2011). Using nonsense-mediated RNA decay microarrays in combination with array comparative genomic hybridization, it was found that the EphB2 gene in the PC metastatic cell line DU145 was completely inactivated (biallelic inactivation). The introduction of wild-type EPHB2 remarkably decreased clonogenic growth of DU145 cells (Huusko et al., 2004). Moreover, Morales et al. found that EphB2 expression was inversely associated with PC cell aggressiveness. EphB2 silencing promoted the proliferation of PC cells and simultaneously induced *de novo* lipid droplet (LD) accumulation in both nuclear and cytoplasmic compartments. A DGAT1-specific inhibitor (A-922500) suppressed LD accumulation induced by EphB2 loss (Morales et al., 2021). However, another study reported that the upregulation of EphB2 and Src Pathways were correlate with advanced PC. After dasatinib treatment or siRNAs knockout of Src or EphB2, the epidermal growth factor receptor (EGFR) dynamics, cell motility, and invasive capabilities of PC cells were significantly reduced. Additionally, the upregulation of partial EphB2 and Src pathways predicted poor prognosis in PC patients (Liu et al., 2019), but the paradoxical results need more thorough investigation. In addition, EphB2 was found to play a crucial role in familial PC. Loss of function mutations in the EphB2 were accompanied by an increased risk of PC development (Kittles et al., 2006).

### EphB2 in Colorectal Cancer

EphB2 has been proven to be a direct transcription target of T-cell factor (TCF)/β-catenin and it was expressed at high levels in colon premalignant lesions (Batlle et al., 2002; van de Wetering et al., 2002). However, EphB2 expression was decreased in colorectal cancer (CRC). Fu et al. revealed that c-Rel serves as a transcriptional inhibitor of EphB2 and plays a positive role in EphB2 downregulation in CRC (Fu et al., 2009). Downregulation of EphB2 expression promoted the progression of CRC (Batlle et al., 2005; Oshima et al., 2008; Noberini and Pasquale, 2009) and was associated with more advanced CRC, poorer differentiation, poorer overall survival and disease-free survival (Jubb et al., 2005; Guo et al., 2006). Moreover, inactivation of EphB2 has been demonstrated to facilitate tumorigenesis caused by APC mutations in the colorectum of APC^Min/+^ mice, indicating that EphB2 acts as a tumor suppressor in the large intestine (Batlle et al., 2005; Cortina et al., 2007). Consequently, although upregulated by TCF/β-catenin signaling, EphB2 inactivation seems to be a crucial requirement for CRC progression. The potential mechanism of EphB2 inactivation was considered to be genetic and epigenetic changes including aberrant promoter methylation (Alazzouzi et al., 2005), loss of heterozygosity (Oba et al., 2001; Lefeuvre et al., 2009), and/or gene mutations (Huusko et al., 2004; Alazzouzi et al., 2005; Davalos et al., 2007). Furthermore, overexpression of EphB2 inhibited CRC cell growth and activation of EphB2 receptor reduced CRC cell migration (Guo et al., 2006; Senior et al., 2010). Yet, EphB2/ephrin signaling was able to suppress CRC expansion and invasion via repulsive mechanisms (Lugli et al., 2005; Okumura et al., 2017; Evergren et al., 2018).

### EphB2 in Breast Cancer

EphB2 was reported to be expressed in benign tissues, but it was significantly upregulated in breast cancer, especially in invasive and metastatic carcinomas (Wu et al., 2004; Chukkapalli et al., 2014). High EphB2 expression was correlated with poor overall survival in breast cancer patients (Wu et al., 2004; Husa et al., 2016; Ebrahim et al., 2021). However, paradoxically, although EphB2 expression induces autophagy and apoptosis, it was also found to promote cell invasion (Chukkapalli et al., 2014). To explain this duality, Kandouz et al. proposed that EphB2 stimulates autophagy, which, conversely, can promote apoptosis or invasion depending on the context. In normal circumstances, autophagy facilitates apoptosis, but when the apoptosis of cancer cells is blocked, autophagy promotes invasion (Chukkapalli et al., 2014). This mechanism could interpret the contradictory role of EphB2 receptor in breast cancer, but this theory requires further research. Furthermore, the EphB2 gene was identified as a novel transforming growth factor (TGF)-β target that is important for the TGF-β3-mediated migration and invasive of breast cancer cells, and its transcriptional activation by TGF-β3 was also suppressed by p53 (Lam et al., 2014). However, how p53 inhibits TGF-β3-induced EphB2 expression requires additional research.

### EphB2 in Hepatocellular Carcinoma

EphB2 expression was found to be stepwise increased from normal liver tissues to cirrhotic liver tissues and to hepatocellular carcinoma (HCC) tissues and associated with poor prognosis (Mimche et al., 2015b; Butler and Schmidt, 2015; Leung et al., 2021). Moreover, knockout of endogenous EPHB2 showed reduced tumor growth in mice. Interestingly, EphB2 was significantly upregulated in the established sorafenib-resistant PDTXs. EphB2^High^ HCC cells were found to have enhanced the traits of liver cancer stem cells (CSCs). Mechanistically, T cell factor-1 (TCF1) regulated the expression of EphB2 through promoter activation to form a positive Wnt/β-catenin feedback loop, thereby regulating cancer stemness and drug resistance. Targeting EphB2 with rAAV-8-shEphB2 inhibited HCC tumor development and obviously sensitized HCC cells to sorafenib in an HCC immunocompetent mouse model (Leung et al., 2021). Taken together, targeting the TCF1/EphB2/β-catenin pathway may act as a promising therapeutic strategy for HCC treatment.

### EphB2 in Pancreatic Cancer

EphB2 was reported to be highly expressed in pancreatic cancer tissues and associated with shortened survival (Lu et al., 2012; Chen et al., 2019). Multivariate analyses showed that EphB2 was an independent prognostic factor in human pancreatic cancer. The overexpression of EphB2 and ephrin-B2 significantly increased the incidence of higher degree of pain, lymph node metastasis, and advanced classification of T factor. Moreover, in the presence of high EphB2 expression, elevated ephrin-B2 levels can cause a more aggressive tumor phenotype (Lu et al., 2012). However, another study reported that silencing EphB2 accelerated pancreatic cancer growth by promoting cell proliferation through triggering G1/S phase transition, which depended on a CyclinD1/CDK6 cell-cycle regulated signal. Similarly, inhibiting EphB2 also reduced the apoptosis of CFPAC-1 cells by upregulating Bcl-2 expression. Furthermore, the high expression of EphB2 indicated a better response rate to Qingyihuaji formula (QYHJ) treatment in pancreatic cancer CFPAC-1 cells (Hua et al., 2011; Hua et al., 2014), but the contradictory results need more thorough research. In addition, QYHJ showed an obvious effect against the gemcitabine (GEM) resistant pancreatic cancer, which probable by inhibiting cell migration, increasing the mRNA expression of lncRNA AB209630, and decreasing the mRNA levels of EphB2, miR-373, and NANOG (Chen et al., 2019).

### EphB2 in Squamous Cell Carcinoma

EphB2 overexpression was detected in head and neck squamous cell carcinoma (HNSCC) patient and correlated with poor patient survival. Functional experiments demonstrated that the expression of EphB2 in small extracellular vesicles (SEVs) regulated HNSCC angiogenesis both *in vivo* and *in vitro*, and EphB2 carried by SEVs induced ephrin reverse signaling through phosphorylation of ephrin-B and STAT3. A STAT3 inhibitor significantly reduced SEV-induced angiogenesis (Sato et al., 2019). Cutaneous squamous cell carcinoma (CSCC)-derived cell lines and tumor tissues were reported to express increased levels of EphB2 mRNA. Knockdown of EphB2 expression inhibited growth and vascularization of CSCC tumors *in vivo* and inhibited proliferation, invasion, and migration of CSCC cells (Farshchian et al., 2015). In the human CSCC cell line A431, silencing of EphB2 also induced epithelial-mesenchymal transition (EMT)-like morphological changes accompanied by an obvious upregulation of EMT-associated genes such as zinc finger E-box binding homeobox 1/2. And EphB2 plays a crucial role in facilitating the anchorage-independent growth of A431 cells by the suppression of EMT (Inagaki et al., 2019). At the same time, activation of EphB2 signaling by ephrin-B2-Fc promoted invasion of CSCC cells and stimulated production of matrix metalloproteinase-13 (MMP13) and MMP1 (Farshchian et al., 2015). Moreover, treatment of CSCC cell lines with dasatinib effectively suppressed phosphorylation of endogenous EphB2, p38 MAPK, and Src, and then inhibited phosphorylation of ERK1/2. Silencing of EphB2 expression partly rescued CSCC cells from the inhibition of dasatinib on cell viability (Farshchian et al., 2017). Furthermore, *in vitro* experiments, EphB2 small-molecule inhibitors obviously inhibited CSCC cell proliferation, migration, invasion, and induced apoptosis. In a xenograft model, EphB2 small-molecule inhibitors induced morphological changes in the EMT, thereby affecting the progression of CSCC (Li and Zhang, 2020).

### EphB2 in Gliomas

EphB2 expression was reported to be significantly higher in gliomas than in normal brain tissues and was correlated with tumor grade (Nakada et al., 2004). overexpression of EphB2 inhibited cell adhesion and promoted cell invasion in glioma tissues and cells (Nakada et al., 2004; Nakada et al., 2005). Mechanistic investigations demonstrated that epigenetic silencing of miR-204 increased EphB2 expression in glioma cells and promoted EphB2-mediated invasion and migration (Ying et al., 2013). Additionally, overexpression of EphB2 decreased the capability of miR-128 to facilitate cell-cell adhesion. The wound-healing assay revealed that miR-128 obviously suppressed cell migration by EphB2 (Lin et al., 2013).

In glioblastoma multiforme (GBM), EphB2 overexpression correlated to poor overall survival in GBM patients. CircMELK could upregulate EphB2 expression by sponging miR-593, thereby promoting the proliferation, invasion, migration, and glioma stem cell (GSC) maintenance of GBM cells (Zhou et al., 2021). Moreover, Qiu et al. reported that EphB2 expression was upregulated in GBM cells under hypoxia and the stabilization of EPHB2 by hypoxia required the participation of hypoxia-inducible factor-2α (HIF-2α). The overexpression of EphB2 enhanced the invasion capability of GBM through the phosphorylation of paxillin under hypoxic conditions (Qiu et al., 2019). However, another study reported that focal adhesion kinase (FAK) activation mediated EphB2-induced actin cytoskeleton organization, focal adhesion formation, and ultimately caused GBM neurosphere cell migration, but EphB2 expression suppressed neurosphere cell proliferation (Wang et al., 2012). The phenomenon that EphB2 has both anti-proliferative and pro-migratory effects *in vivo* may reflect the migration/proliferation dichotomy of GBM, whereas its underlying molecular mechanisms are largely unclear (Giese et al., 1996; Giese et al., 2003).

EphB2 in Medulloblastoma EphB2 was reported to be overexpressed in medulloblastoma patient samples than in normal cerebellum (Sikkema et al., 2012; Coudière Morrison et al., 2013; Bhatia et al., 2017). EphB2 knockdown combined with radiation exposure induced G2/M cell cycle arrest, reduced clonogenic survival fractions, inhibited medulloblastoma cell viability, and reduced medulloblastoma cell invasion (Bhatia et al., 2017). The efficacy of this combined modality can be further tested in other pre-clinical models. Moreover, Sikkema et al. reported that stimulation with ephrin-B1 resulted in a significant decrease in cell adhesion *in vitro* and an increase in invasion ability of medulloblastoma cells expressing high levels of EphB2. Furthermore, analysis of signal transduction found that Erk, mTOR, and p38 are downstream signaling mediators, which may induce the ephrin-B1 phenotype (Sikkema et al., 2012).

### EphB2 in Cervical Cancer

EphB2 expression was reported to be upregulated and significantly associated with cancer progression and stage malignancy in the cervical cancer (CC) (Narayan et al., 2007; Gao et al., 2014), and the overexpression of EphB2 induced CC cells to undergo epithelial-mesenchymal transition (EMT) and acquire stem cell-like properties by activating the R-RAS pathway (Gao et al., 2014). Moreover, Duan et al. reported that EphB2 was a direct target of miR-204 and knockdown of EphB2 obtained the inhibitory effect of miR-204 mimic on the proliferation, migration, and invasion of CC cells (Duan et al., 2018).

### EphB2 in Malignant Mesothelioma

EphB2 was reported to be overexpressed in malignant mesothelioma (MM) cell lines and tumor tissues. EphB2 inhibition was involved in the decrease of cell proliferation, invasion, migration, and colony formation and the increase of apoptotic cells. Silencing the EphB2 expression is related to the decrease of cell proliferation, migration, invasion and colony formation and the increase of apoptotic cells. Moreover, targeting EphB2 knockout in H2595 and HP-1 cell lines increased the expression of downstream targets such as caspase-2 and caspase-8, whereas vascular endothelial growth factor (VEGF) and matrix metalloproteinase (MMP-2) had decreased expression (Goparaju et al., 2013).

### EphB2 in Bladder Cancer

EphB2 expression was reported to be absent or decreased in bladder cancer tissues, compared to the normal bladder tissues (Li et al., 2014; Lee et al., 2021). Low expression of EphB2 was significantly correlated with advanced clinical stage, muscular invasion, higher tumor grade, and a high incidence of cystectomy. Moreover, *in vitro* studies demonstrated that EphB2 inactivation promoted cell proliferation, motility, and invasion of bladder cancer, implying that EphB2 loss was involved in tumor metastasis and invasion of bladder cancer (Lee et al., 2021).

### EphB2 in Other Tumors

EphB2 overexpression was detected in lung adenocarcinoma (AC) tissues. High expression of EphB2 was remarkably associated with poor overall survival of lung AC patients (Zhao et al., 2017). EphB2 was found to be overexpressed in ovarian carcinoma and correlated with poor prognosis (Wu et al., 2006). Moreover, EphB2 expression was reported to be increased in cholangiocarcinoma (CCA) tissues. High expression of EphB2 was remarkably associated with CCA patient’s metastasis status. EphB2 suppression by siRNA obviously decreased CCA cell migration through reducing the phosphorylation level of paxillin and focal adhesion kinase (FAK) (Khansaard et al., 2014). Furthermore, EphB2 had significantly lower expression in Wilms tumor tissues compared to normal kidney tissues, but its role in Wilms tumor requires further research (Chetcuti et al., 2011).

## Prospects and Conclusion

EphB2 is a significant member of the Eph receptor family, which was thought to be distributed on tumor cells and endothelial cells in previous researches (Salvucci et al., 2006; Wang et al., 2012). Recently, as EphB2 was found to be expressed on some immunocytes such as monocytes, T cells, and B cells, increasing researches have reported on the roles of EphB2 in immunity (Alfaro et al., 2008; Braun et al., 2011; Yu et al., 2014). Forward EphB2 signaling induced by the specific binding of ephrin-B1/B2 and EphB2 could promote monocyte activation and T-cell migration (Alfaro et al., 2008; Braun et al., 2011). In addition, transdifferentiation of human monocytes into macrophages was correlated with increased expression of EphB2, and exposure of monocytes to immobilized ephrinB2 led to phosphorylation of receptors, followed by increased expression of proinflammatory chemokines such as monocyte chemotactic protein-1/CCL2 and interleukin-8 (Braun et al., 2011). Yu et al. reported that EphB2 was involved in the activation of human naive B-cell via Notch1 and Src-p65 signaling pathways and was regulated by miR-185 (Yu et al., 2014). In this study, they used Western blot to test the expression of EphB2 on B cells, and used EphB2 siRNA interference in human B cells from healthy volunteers to evaluate the roles of EphB2 in immunoglobulin (Ig) production, cytokine secretion, and B-cell proliferation. Their demonstrated that EphB2 was scattered on naive B cells and its expression was up-regulated on activated B cells. IgG production (decreased by 26%, *p* < 0.05), TNF-α secretion (decreased by 40%, *p* < 0.01), and B-cell proliferation (decreased by 22%, *p* < 0.05) were decreased concordantly with the down-regulated EphB2 expression. Subsequently, they found that miR-185 directly targeted EphB2 mRNA and inhibited its expression. Moreover, miR-185 overexpression suppressed B-cell activation and miR-185 inhibitor promoted B-cell activation. Furthermore, abatement of EphB2 via EphB2 siRNA or miR-185 mimics attenuated the activation of Notch1 and Src-p65 signaling pathways in human B cells (Yu et al., 2014). In conclusion, it can be speculated that EphB2 might be involved in tumor immunity and this issue certainly worthy further investigation in future research. In this review, we first systematically summarized and discussed the roles of EphB2 in cancer, as well as listed researches that may deepen our understanding of how it regulates cancer progression. Overall, EphB2 serves as a tumor promoter in most cases, facilitating tumor cell proliferation, invasion, and migration through different signaling pathways. However, EphB2 expression and its specific functions in gastric cancer and prostate cancer are controversial and need to be further studied. Moreover, the relationship between EphB2 expression and clinicopathological features was summarized. In detail, the abnormal expression of EphB2 was remarkably associated with clinicopathological features, including overall survival, disease-free survival, lymph node metastasis, histological differentiation, tumor grade and stage, reflecting its potential value as a sensitive and effective biomarker for cancer diagnosis, prognosis, and therapy. Furthermore, The Eph receptor family is an attractive tumor therapeutic target. Previous studies have developed a peptide, monoclonal antibody or small molecule against EphB2 in an attempt to prevent its activation (Mao et al., 2004; Koolpe et al., 2005; Toledo-Sherman et al., 2005), which may be used as a potential treatment for cancer. Accordingly, deepening the understanding of the structure, biogenesis, and molecular mechanisms of EphB2 will provide valuable information for functional research and improving the efficiency of rational drug design.
